# Analysis of a four generation family reveals the widespread sequence-dependent maintenance of allelic DNA methylation in somatic and germ cells

**DOI:** 10.1038/srep19260

**Published:** 2016-01-13

**Authors:** Aifa Tang, Yi Huang, Zesong Li, Shengqing Wan, Lisha Mou, Guangliang Yin, Ning Li, Jun Xie, Yudong Xia, Xianxin Li, Liya Luo, Junwen Zhang, Shen Chen, Song Wu, Jihua Sun, Xiaojuan Sun, Zhimao Jiang, Jing Chen, Yingrui Li, Jian Wang, Jun Wang, Zhiming Cai, Yaoting Gui

**Affiliations:** 1 Shenzhen Key Laboratory of Genitourinary Tumor, National-Regional Key Engineering Technology Laboratory for Clinical Application of Cancer Genomics, Shenzhen Second People’s Hospital, the First Affiliated Hospital of Shenzhen University, Shenzhen 518035, China; 2 Guangdong and Shenzhen Key Laboratory of Male Reproductive Medicine and Genetics, Institute of Urology, Peking University Shenzhen Hospital, Shenzhen PKU-HKUST Medical Center, Shenzhen 518036, China; 3 The Genome Institute, Washington University in St Louis, Missouri 63108, USA; 4 Luohu People’s Hospital, Shezhen 518001, China; 5 BGI-Shenzhen, Shenzhen 518083, China

## Abstract

Differential methylation of the homologous chromosomes, a well-known mechanism leading to genomic imprinting and X-chromosome inactivation, is widely reported at the non-imprinted regions on autosomes. To evaluate the transgenerational DNA methylation patterns in human, we analyzed the DNA methylomes of somatic and germ cells in a four-generation family. We found that allelic asymmetry of DNA methylation was pervasive at the non-imprinted loci and was likely regulated by *cis*-acting genetic variants. We also observed that the allelic methylation patterns for the vast majority of the *cis*-regulated loci were shared between the somatic and germ cells from the same individual. These results demonstrated the interaction between genetic and epigenetic variations and suggested the possibility of widespread sequence-dependent transmission of DNA methylation during spermatogenesis.

DNA methylation is one of the most important epigenetic marks that regulate the normal development and differentiation processes in human, and disturbance of DNA methylation is associated with various diseases^1^,^2^,^3^. The homologous chromosomes from diploid mammals usually show different methylation patterns (allele-specific DNA methylation, ASM) at specific loci including the imprinting regions and X-chromosomes in females^1^. Recent studies indicated that such type of allelic asymmetry of DNA methylation was likely to be more prevalent at non-imprinted loci on autosomes, and the methylation pattern of these loci was generally suspected to be associated with DNA sequence variants acting in *cis*^4^,^5^,^6^. However, it is largely unclear about the extent of allelic asymmetry of DNA methylation across the human genome. Moreover, comparison of the methylation patterns of the ASM loci across generations or between somatic and germ cells had not been fully investigated previously.

To understand the possible roles of *cis*-acting genetic variants in establishing the methylation statuses of the two alleles at a locus over generations, we obtained peripheral blood samples (PBSs) and sperm samples from a four-generation family. To identify the genomic loci whose methylation statuses were likely to be sequence dependent, we performed a genome-wide survey of the degree of ASM by combining the data generated by the methylated DNA immunoprecipitation sequencing (MeDIP-seq) technology and the Illumina SNP genotyping platform. The methylation patterns of the SNP tagged ASM loci were compared between the somatic and germ cells. Sanger sequencing of the cloned bisulfite PCR products was also performed on a number of genomic regions to validate the candidate ASM events.

## Results

### Data production and reads mapping

**Figure 1:**
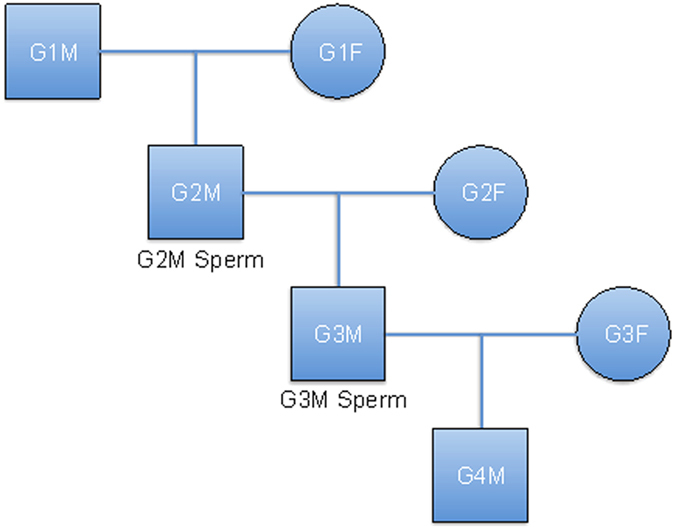
The pedigree structure of the family analyzed in the study. G, generation; M, male; and F, female. Methylation analysis was performed for the PBSs from all the seven individuals and the two sperm samples from G2M and G3M.

Seven PBSs and two sperm samples were collected from the family members (Fig. 1) and genomic DNA was extracted from each sample for sequencing and genotyping analyses. After library preparation, the adaptor ligated DNA was immunoprecipitated by the anti-5mC antibody and pair-end or single-end reads with an average length of 49 bp were generated with the Illumina sequencing platform. In total, we generated more than 67 million MeDIP-seq reads per sample, of which at least 83% could be aligned to the human reference genome (build NCBI 36) (Supplementary information, Table S1). On average, for each of the nine samples, about 58% (ranged 52-71%) of all the autosomal CpG sites were estimated to be covered by at least one extended reads with high mapping qualities (MAQ score ≥10) (Supplementary information, Figure S1)^7^. As indicated in previous methylome analyses^8^, the amount of sequence data or CpG coverage achieved in our study was sufficient to provide reliable genome-wide methylation profiles.

### MeDIP-seq detects X-chromosome inactivation

Promoter DNA methylation has a well-established role in the inactivation of genes on the X-chromosomes in females. We divided the promoters of X-chromosomal genes into two subtypes according to whether they were associated with adjacent CpG islands (CGIs) and observed that the normalized methylation signals for the CGI-associated promoters were significantly higher on the X-chromosomes than on the autosomes in PBSs from the females (all *P* < 0.05; Supplementary information, Figure S2). On the other hand, as shown in previous studies^9^, no significant difference in the methylation signals was observed for the promoters without CGIs in females. The methylation patterns on the X-chromosomes indicated that we could use MeDIP-seq read signals to measure the methylation levels in our samples.

### Identification of ASM events based on data from sequencing and SNP arrays

**Figure 2:**
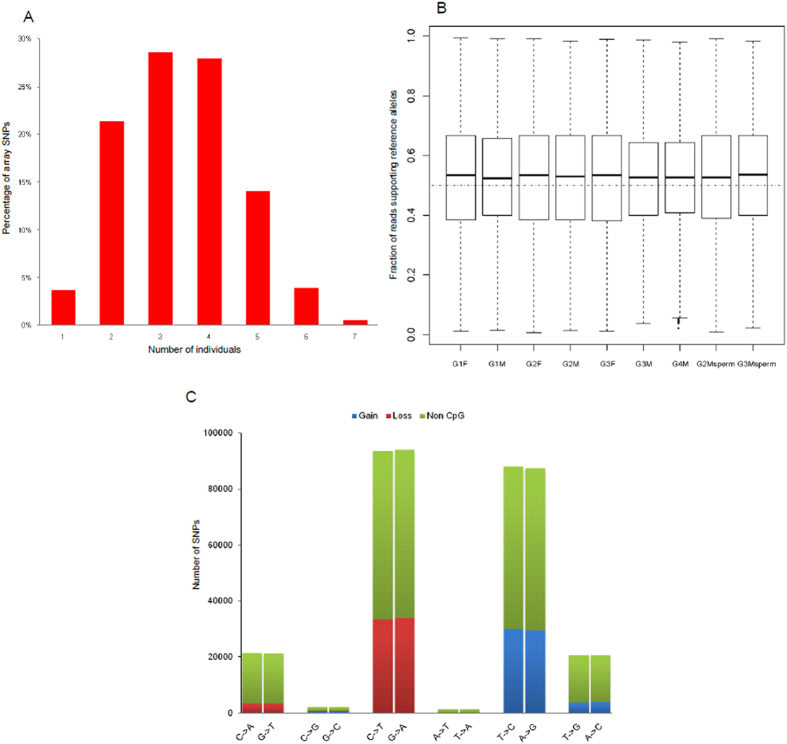
The distribution patterns of SNPs genotyped by the Illumina arrays. (**A**) Percentages of heterozygous SNPs that were shared by different numbers of individuals. (**B**) Fractions of reads supporting reference alleles for the heterozygous sites that were covered by at least 10 reads. (**C**) The mutation spectrum of SNPs genotyped by the Illumina arrays. The ratio of the number of SNPs resulting in loss of a CpG site to the number of SNPs resulting in gain of a CpG site was 1.10.

To identify the SNP tagged ASM events, we first focused on the heterozygous SNPs that were genotyped by the Illumina SNP arrays. Of the ~50 k heterozygous array SNPs, 96.4% were shown to be heterozygous in at least two individuals (Fig. 2A), which provided a large number of SNP candidates for the identification of ASM associated SNPs. We assumed that, if the two alleles at a genomic region were differentially methylated within a homogenous cell population, the hypermethylated alleles should be over-represented in the MeDIP-seq data. Thus, we considered a heterozygous SNP to be a ASM associated tag SNP if (i) it was covered by sufficient read depth (≥10×) and (ii) showed significant over-representation of one allele in the MeDIP-seq sequencing data (FDR < 0.1). To further reduce the false positives, we also required that (i) the SNP tagged ASM events should be supported by at least two PBSs and (ii) the trend of allelic preference should be consistent across different individuals for a given ASM event (Materials and Methods).

As a proof of the above concept for identifying the ASM events, we calculated the percentage of MeDIP reads supporting the reference allele (the allele reported by the human reference genome at a given SNP site) for each heterozygous SNP that was covered by 10× or greater in the PBSs. Generally, our MeDIP-seq approach did not show obvious strand bias of sequencing reads for the majority of heterozygous SNPs. The mean values of the percentages of reference allele supporting reads for the heterozygous SNPs were only slightly deviated from 0.5 (Fig. 2B). Further analysis of the mutation spectrum of the array SNPs and the fractions of reference allele supporting reads for the non-CpG SNPs revealed that this slight deviation might be partly due to the fact that there were more heterozygous SNPs resulting in loss of CpG sites than those resulting in gain of CpG sites (Fig. 2C and Supplementary Information, Figure S3). Of the ~100 array SNPs that were considered to be associated with ASM events, all showed significant over-representation of one of the alleles (Supplementary information, Table S2 and Figure S4A).

Various studies had shown that most of ASM events at the non-imprinted loci could be defined as methylation quantitative trait loci (mQTL) and were most likely to be linked to *cis*-acting regulatory variants^10^,^11^,^12^. We correlated the array SNP genotype data with MeDIP-seq data in our study by normalizing the read count data for all the SNPs across all the seven PBSs. SNPs that showed the same genotype in all seven individuals were filtered out from this analysis. We observed that the majority of ASM associated tag SNPs showed a genotype-dependent methylation pattern in the PBSs as measured by the mean number of reads supporting each genotype (Supplementary information, Figure S4B). In general, the heterozygotes had lower methylation levels than the homozygotes of the methylated alleles while had higher methylation levels than the homozygotes of the demethylated alleles. Therefore, our data supported that most of the ASM events identified by our analysis were likely mQTLs linked with *cis*-acting genetic factors.

### Identification of additional ASM from other genomic regions

Nearly all the published studies that documented ASM events in human were based on targeted methods that can only analyze the methylation patterns of predefined genomic regions or loci^4^,^5^,^13^. Although bisulfite sequencing is the gold standard technique for studying DNA methylation, bisulfite treatment of genomic DNA obscures C- > T/G- > A SNPs, which account for one of the largest groups of human SNPs (Fig. 2C). Therefore, it was difficult to get a full spectrum of the genetic variants if SNP calling was based on the bisulfite sequencing data solely. In our study, we took advantage of the genome-wide nature of MeDIP-seq approach and extrapolated the above findings from the array SNPs to the rest of human genome by calling SNPs from the MeDIP-seq data (Materials and Methods). We observed a high genotype concordance rate between the SNPs called by MeDIP-seq and those genotyped by the Illumina SNP array (Supplementary information, Figure S5), which indicated that most of the SNPs identified by MeDIP-seq were likely to be true genetic variants. Since all the SNPs genotyped by the Illumina SNP array are common genetic polymorphisms that have been deposited in public databases, we further refined the genetic variant set called out by MeDIP-seq to those polymorphisms that had been reported in dbSNP132 or the 1000 Genome Project to reduce the false positives.

**Figure 3:**
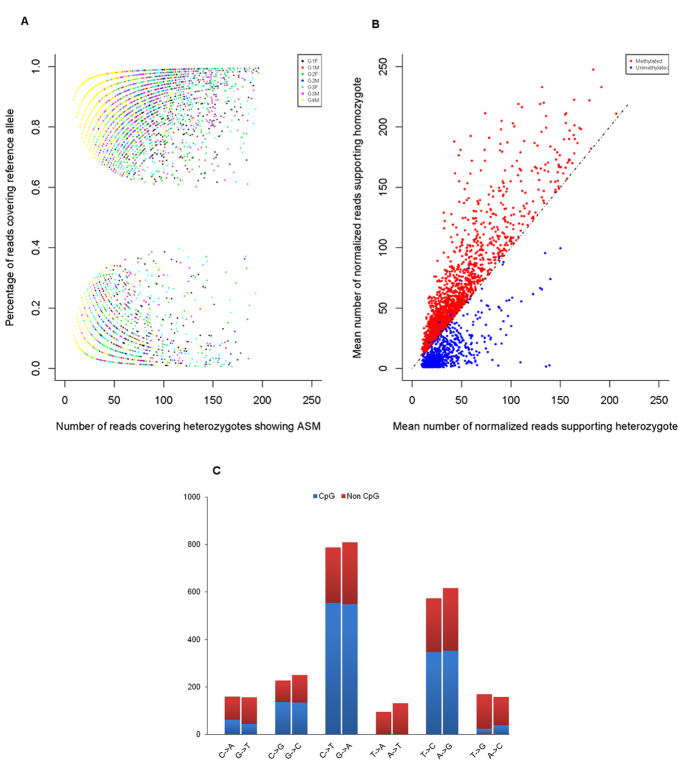
The distribution patterns of the ASM associated tag SNPs that were identified by MeDIP-seq. (**A**) Fractions of reads supporting the reference alleles of the ASM tag SNPs. (**B**) The mean numbers of reads supporting the different genotypes of the ASM tag SNPs. (**C**) The mutation spectrum of the ASM tag SNPs.

We applied the same analysis strategy described above and identified ~ 4, 000 ASM events across the human genome. These ASM events showed significantly allelic asymmetry of DNA methylation and also showed a strong genotype-dependent effect on their methylation levels (Fig. 3A,B). Previous studies reported that a substantial proportion of ASM events was contributed by CpG-SNPs in human^13^,^14^. We also found that over half of the ASM events in our study were closely linked to SNPs disrupting the CpG dinucleotide sites (Fig. 3C). Interestingly, we also noticed that the C- > T/G- > A SNPs represented the largest group of ASM associated genetic variants, which could be easily missed by the bisulfite sequencing approaches.

### Validation of ASM events by Sanger sequencing of the cloned bisulfite PCR products

**Figure 4:**
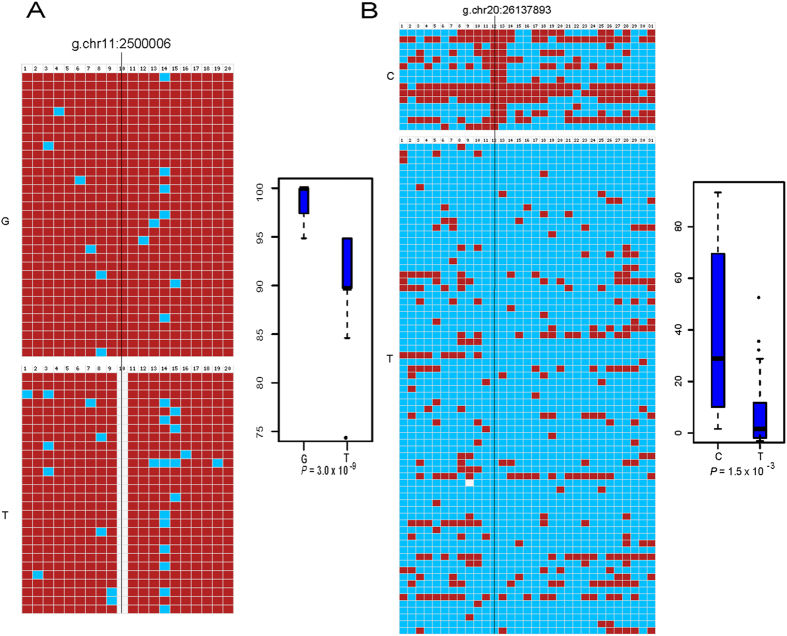
ASM events validated by Sanger sequencing of the cloned bisulfite PCR products. The sequencing reads are grouped according to the alleles at the tag SNP positions. The small squares represent the CpG sites on individual sequencing reads. The methylated CpGs are marked in dark red color, the demethylated CpGs are marked in light blue color and CpGs affected by SNPs are marked in white color. The positions of the tag SNPs are also indicated. The distributions of the methylation rates of individual sequencing reads across all CpG sites are shown in the box plots. The validation results of bisulfite sequencing for ASM regions overlapped with SNPs g.chr11:2500006 (**A**) and g.chr20:26137893 (**B**).

Ten candidate ASM events with different levels of CpG contents and different levels of MeDIP-seq signals were randomly selected for validation with bisulfite PCR amplification, cloning and Sanger sequencing in the PBSs. Bisulfite sequencing reads from individual clones were grouped according to their alleles at the tag SNP sites and bisulfite sequencing reads from different individuals with the same alleles were merged together. The mean methylation rates between the two different alleles were compared by *t*-test. All the potential ASM regions detected by our MeDIP-seq approach were confirmed by the results of cloned bisulfite PCR sequencing (Fig. 4 and Supplementary Information, Figure S6). Interestingly, although bisulfite treatment obscures the C- > T tag SNPs in forward strand reads, we selected one candidate ASM region overlapped with a C- > T SNP at the non-CpG site for validation with bisulfite sequencing. We found that the methylation levels of individual cloned sequencing reads varied substantially from nearly unmethylated to fully methylated but no SNP signal could be detected in the cloned sequencing data (Supplementary Information, Figure S6D). For cloned sequencing, individual sequencing reads were derived from different alleles and this C- > T SNP associated ASM event represented an important kind of ASM events that could not be identified by bisulfite sequencing directly and substantiated the necessity to use methylation data without bisulfite conversion for the identification of ASM. Overall, these results indicated that acceptable sensitivities and reliabilities could be achieved when we used the MeDIP-seq data to identify ASM events.

### Functional annotation of the ASM tag SNPs

Only a small percentage of the ASM associated tag SNPs was located in genomic regions with well-studied regulatory roles, including promoters, CGIs and CGI shores, while the majority of them were located in other genomic regions without well-defined functional roles (Table 1). However, comparing with the frequency distribution patterns of all the SNPs identified by MeDIP-seq, we found that the ASM tag SNPs were significantly over-represented in the CGIs and were significantly under-represented in the genic regions (both *P* < 2.2 × 10^−16^, Table 1). These findings may imply that ASM events were more likely to occur in genomic regions under less selection pressure^13^. To further validate this possibility, we analyzed the evolutionary constraint on the ASM associated polymorphic sites by using the phyloP conservation scores as the measurement of inter-species conservation. Student’s t-test was used to compare the degree of conservation between different sets of SNPs. We found that the ASM tag SNPs were significantly less conserved than the SNP sets that were genotyped by the Illumina arrays or were identified by the MeDIP-seq approach (Supplementary information, Figure S7). This trend of association between phyloP scores and ASM statuses remained significant (*P* = 3.45 × 10^−12^) even when we further corrected the potential effects of read depths by regression analyses.

### Comparing the methylation pattern of the ASM loci between PBSs and sperm samples

Recent studies had proved that the *cis*-regulated ASM events identified in one type of somatic tissue could be detected in other tissue types from the same individual, raising the possibility that the allelic methylation pattern might be present at quite early stages during embryonic development^13^. Nevertheless, there was no direct or indirect evidence demonstrated whether the allelic asymmetry of DNA methylation in somatic tissues could be shared by the germ cells despite of the extensive round of epigenetic reprogramming during gamete formation. Thus, we further evaluated the extent of allelic asymmetry of DNA methylation at genomic loci that showed ASM in the PBSs in their matched sperm samples. We observed that a small number of ASM tag SNPs either had a quite low coverage of read depth or showed no obvious trend of allelic preference in the MeDIP-seq data in the sperm samples (Supplementary Information, Figure S8), suggesting that epigenetic marks at these genomic loci had been erased or reprogrammed during spermatogenesis. Interestingly, we also found that over 70% of the tag SNPs that were linked to ASM events in PBSs also showed a consistently asymmetric pattern of DNA methylation in their matched sperm samples (Supplementary Information, Figure S8). These observations may suggest that the sequence dependent mechanism of maintaining methylation patterns was also likely to be effective during spermatogenesis for a number of genomic loci.

## Discussion

It has becoming increasingly clear that asymmetry of DNA methylation between the two homologous chromosomes in human is pervasive at non-imprinted loci and is usually regulated by *cis*-acting genetic variants^4^,^5^,^6^,^13^. Because of the differences in methylation profiling techniques and statistical analyses, the estimated degree of ASM across human genome varied substantially between studies. To explore the genetic-epigenetic interactions, it is necessary to analyze the genetic variants and DNA methylation patterns simultaneously. Without bisulfite treatment which may obscure C- > T/G- > A SNPs, our study demonstrated the possible application of MeDIP-seq to identify ASM events across human genome. However, the number of SNPs identified in this way is limited by the sequencing depths and more ASM events can be identified with the increase of sequencing depths. In our analysis, the numbers of MeDIP sequencing reads covering the tag SNPs represented the overall relative methylation levels of the surrounding genomic regions (about 440–640 bp in length), rather than the absolute methylation levels of individual CpG sites. Because the methylation patterns are highly correlated between adjacent CpG sites, it appeared to be more reasonable to identify the ASM events using the region-based method.

The ASM events identified in our study were different from genomic imprinting. The methylation levels of the ASM events at non-imprinted loci were dependent on the genotypes of *cis*-acting variants as opposed to parental origin^15^. In addition, the imprinted loci often showed a biphasic methylation profile (either fully methylated or demethylated) between the two alleles while most of the ASM events at non-imprinted loci showed more subtle changes in methylation levels between the two alleles^5^. In principle, the MeDIP-seq based approach could not be applied to identify the biphasic ASM event as no MeDIP-seq read signal could be detected for the completely unmethylated alleles.

It remains controversial on whether the DNA methylation polymorphisms can be stably maintained between generations in human. In mammals, the two rounds of extensive epigenetic reprogramming during early embryogenesis and germ cell development appear to be effective in preventing the perpetuation of epigenetic variation between generations^16^. A number of studies showed that the methylation statuses of the *cis*-regulated ASM events were almost absolutely dependent on the genotypes of adjacent genetic variants. For a given sequence-dependent ASM event, the allelic asymmetry of DNA methylation could be observed in both unrelated individuals with distinct genetic background and in different tissues from the same individual^13^. These findings suggested the presence of ASM events at quite early embryogenesis stages. In our study, we provided evidence showing that most of the ASM events identified in somatic tissues were also present in the sperm samples, indicating a sequence-dependent maintenance of ASM was also likely to be widespread during spermatogenesis. However, most of the ASM associated SNP were located in the intergenic regions without well-defined functions and were less conserved during mammalian evolution, suggesting that there was selection pressure to maintain DNA methylation between generations during evolution. Nevertheless, we could not exclude the possibility that ASM may potentially play a role in regulating the functions of non-coding RNAs (such as miRNA and lncRNA). For example, the ASM region tagged by the g.chr20:26137893 T- > C SNP is located upstream of miR-663 and the allelic asymmetry of DNA methylation in the miR-663 promoter region was also validated by the cloned bisulfite sequencing data (Fig. 4B). Several previous studies had demonstrated that miR-663 was a candidate tumor suppressor which regulates the expression of multiple target genes including *HRAS, TP53* and *TGFB1* and the expression of miR-663 itself was likely regulated by DNA methylation in multiple cell lines^17^,^18^,^19^. The roles of miR-663 in regulating the differentiation, aging and other developmental processes have not been investigated and more future studies are warranted.

The identification of ASM tag SNPs may also have important implications for the interpretation of association signals from genome wide association studies. It is usually difficult to pinpoint the location of the true *cis*-regulatory genetic variants around the most significant association signals. Since the *cis*-effects of genotypes on DNA methylation are likely to be local and discrete, the ASM tag SNPs may also serve as an effective surrogate for refining the nearby regulatory variants.

## Materials and Methods

### Sample description and preparation

This study was approved by the ethical committees of Shenzhen Second People’s Hospital and Peking University Shenzhen Hospital, and the experiments were carried out in accordance with the principles set out in the WMA Declaration of Helsinki and the NIH Belmont report. All individuals of this four-generation Chinese Han family with no history of genetic disorders signed an informed consent form permitting the collection and use of their blood samples in the study. Semen samples were obtained from G2M and G3M by masturbation into sterile containers after 3–5 days of sexual abstinence and left to liquefy at 37 °C. Genomic DNA was extracted for the PBCs from all the seven family members and for the sperm samples from two male members using the Qiagen DNA Genomic-tip kit according the manufacturer’s instruction (Qiagen, Hilden, Germany).

### Library preparation, immunoprecipitation of methylated DNA and Illumina based sequencing

DNA was fragmented as described in the DNA bisulfite protocol^20^. End repair, base addition and adaptor ligation steps were performed using Illumina’s Paired-End DNA Sample Prep kit following the manufacturer’s instructions. Adaptor-ligated DNA was immunoprecipitated by the anti-5mC antibodies, and the MeDIP products were validated by qPCR using SYBR green mastermix (Applied Biosystems) and primers for positive and negative control regions supplied in the MeDIP kit (Diagenode). The qPCR validation procedures consisted of 95 °C 5 min, followed by 40 cycles 95 °C 15 s and 60 °C 1 min. The MeDIP DNA was purified with ZYMO DNA Clean & Concentrator-5 column following the manufacturer’s instructions and amplified by adaptor-mediated PCR. After excising the amplified DNA to a size range of 220–320 bp on the 2% agarose gel, the amplification quality and quantity were evaluated using an Agilent 2100 Analyzer and DNA 1000 chips. The libraries were sequenced with the Illumina GAII platform and 44 bp single or pair end reads were generated. Image analysis and base calling were performed by the Genome Analyzer Pipeline version 1.3 using default parameters.

### Read mapping

MAQ was used to align the sequencing reads to the UCSC reference human genome (hg18) with the default parameters^7^. After the alignment, only the confidently aligned reads (mapping quality ≥10) were retained for subsequent analysis. The single-end reads were extended to 200 bp long, which represented the individual DNA fragments pulled down in the methylation enrichment experiment. Likewise, both ends of the pair-ended reads were ligated to represent the methylated individual DNA fragments.

### Identification of ASM events

The software package SAM tools was used to generate the pileup file for each sample^21^. All genomic sites covered by less than 10 reads were removed from further analysis. A genomic locus would be called out as a candidate polymorphic site if there were at least one high quality reads supporting the variant allele. To reduce the false positives, we only focused on those candidate variants that had been reported as genetic polymorphisms in the public databases (dbSNP 132 and the 1000 Genome project). Furthermore, we also required that the genetic variants should be transmitted in a way that follows the Mendel’s laws of inheritance. The binominal distribution test with a hypothesized probability of 0.5 was used to identify the SNP sites that showed significant over-representation of only one of the two alleles in the MeDIP-seq data (*P* < 0.05). ASM events fulfilling the following criteria were removed from further analysis to reduce the false positives: (i) the ASM tag SNPs supported by only one PBS and (ii) the tag SNPs showing inconsistent allelic preference across different individuals. False discovery rates (FDRs) were calculated by combining the binominal *P* values of all SNPs from all individuals to address the problem of multiple testing corrections.

### Validation of ASM events by bisulfite sequencing PCR

After quantification, DNA was bisulfite converted using the EpiTect Bisulfite Kit (Qiagen, Hilden, Germany). Bisulfite-specific primers with a minimum length of 18 bp were designed using the online program MethPrimer^22^. All primers were tested for their ability to yield high quality sequences. Bisulfite modified DNA was amplified in a 25 μL reaction with HotStarTaq DNA polymerase (Qiagen, Hilden, Germany). The PCR was performed with initial denaturing at 95 °C for 15 min, followed by 40 cycles at 94 °C for 30 s, annealing at 54 °C for 30 s, extension at 72 °C for 30 s, and a final extension at 72 °C for 10 min. For bisulfite sequencing, PCR products were purified and subcloned into the pGEM-T vector (Promega, Madison, WI, USA). About 20 white clones for each sample were randomly selected. Each clone was sequenced using ABI 3730 to determine the methylation status of CpG sites. Five to seven PBSs were used to validate each of the ten of candidate ASM region with cloned bisulfite sequencing. The cloned sequencing data were analyzed with the BISMA software program^23^.

## Additional Information

**How to cite this article**: Tang, A. *et al.* Analysis of a four generation family reveals the widespread sequence-dependent maintenance of allelic DNA methylation in somatic and germ cells. *Sci. Rep.* **6**, 19260; doi: 10.1038/srep19260 (2016).

## Supplementary Material

Supplementary Dataset

## Figures and Tables

**Table 1:** Functional annotation of the SNPs identified by MeDIP-seq.

	All MeDIP SNPs			ASM tag SNPs		
	CpG	Non CpG	Total	CpG	Non CpG	Total
Promoter	0.5%	0.5%	1.1%	0.7%	0.4%	1.1%
CGI	2.5%	1.5%	3.9%	4.9%	2.7%	7.6%
CGI shore	6.2%	5.4%	11.6%	6.6%	6.4%	13.0%
Genic	22.7%	18.5%	41.2%	19.6%	11.8%	31.4%
Other	21.3%	20.9%	42.2%	22.5%	24.5%	47.0%
Total	53.1%	46.9%	100.0%	54.2%	45.8%	100.0%
