# Supplementary material for: Analysis of a four generation family reveals the widespread sequence-dependent maintenance of allelic DNA methylation in somatic and germ cells
Source: Sci Rep. 2016 Jan 13;6:19260. doi: 10.1038/srep19260 (PMC4713049; doi:10.1038/srep19260)
Supplement: Supplementary Dataset [file srep19260-s1.doc]

**Supplementary Information:**

**Analysis of a four generation family reveals the widespread sequence-dependent maintenance of allelic DNA methylation in somatic and germ cells**

Aifa Tang^1,2*^, Yi Huang^1,3*^, Zesong Li^1,2*^†, Shengqing Wan^4^, Lisha Mou^1,2^, Guangliang Yin^5^, NingLi^5^, Jun Xie^2^，Yudong Xia^5^, Xianxin Li^2^, Liya Luo^1^, Junwen Zhang^2^, Shen Chen^2^, Song Wu^1,3,4^, Jihua Sun^5^, Xiaojuan Sun^1^, Zhimao Jiang^2^, Jing Chen^2^, Yingrui Li^5^, JianWang^5^, Jun Wang^5^, Zhiming Cai^1^, Yaoting Gui^2^†

^1^Shenzhen Key Laboratory of Genitourinary Tumor, National-Regional Key Engineering Technology Laboratory for Clinical Application of Cancer Genomics, Shenzhen Second People's Hospital, the First Affiliated Hospital of Shenzhen University, Shenzhen 518035, China

^2^Guangdong and Shenzhen Key Laboratory of Male Reproductive Medicine and Genetics, Institute of Urology, Peking University Shenzhen Hospital, Shenzhen PKU-HKUST Medical Center, Shenzhen 518036, China;

^3^The Genome Institute, Washington University in St Louis, Missouri 63108, USA;

^4^Luohu People’s Hospital, Shezhen 518001, China;

^5^BGI-Shenzhen, Shenzhen 518083, China.


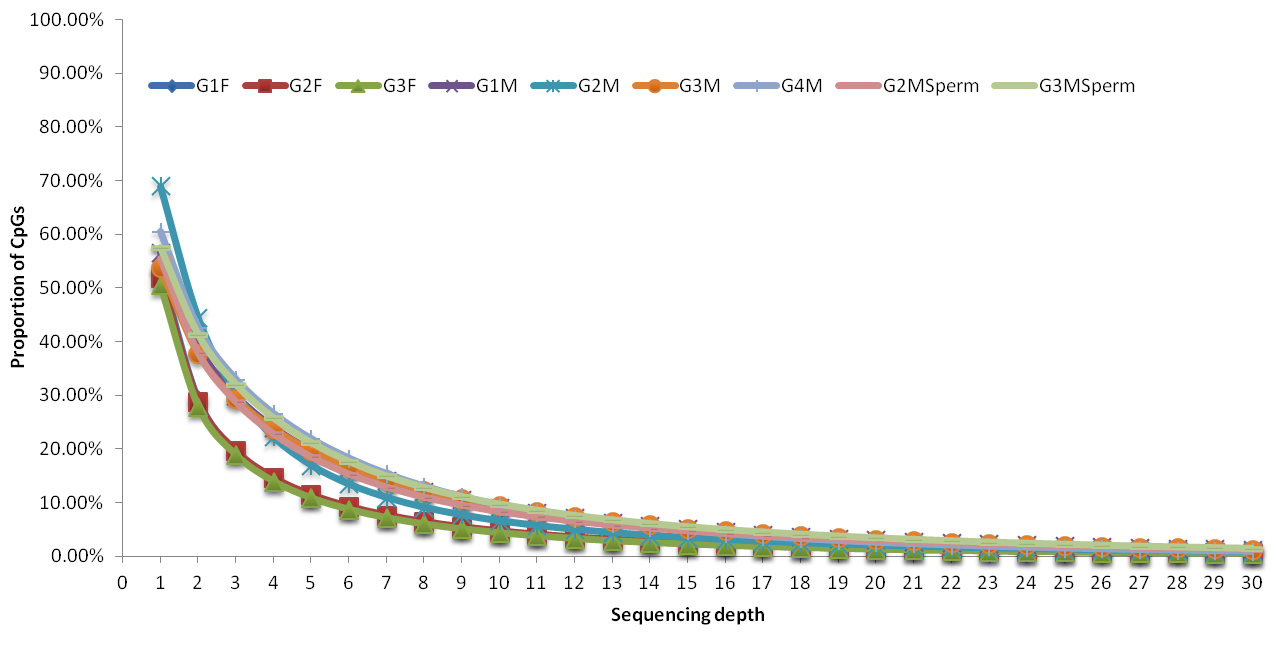


**Figure S1** The genome-wide CpG coverage of all autosomes for each sample. The single-end reads were extended 200bp long, which represents the individual DNA fragments pulled down in the methylation enrichment experiment. Similarly, both ends of the pair-ended reads were ligated to represent the methylated individual DNA fragments.


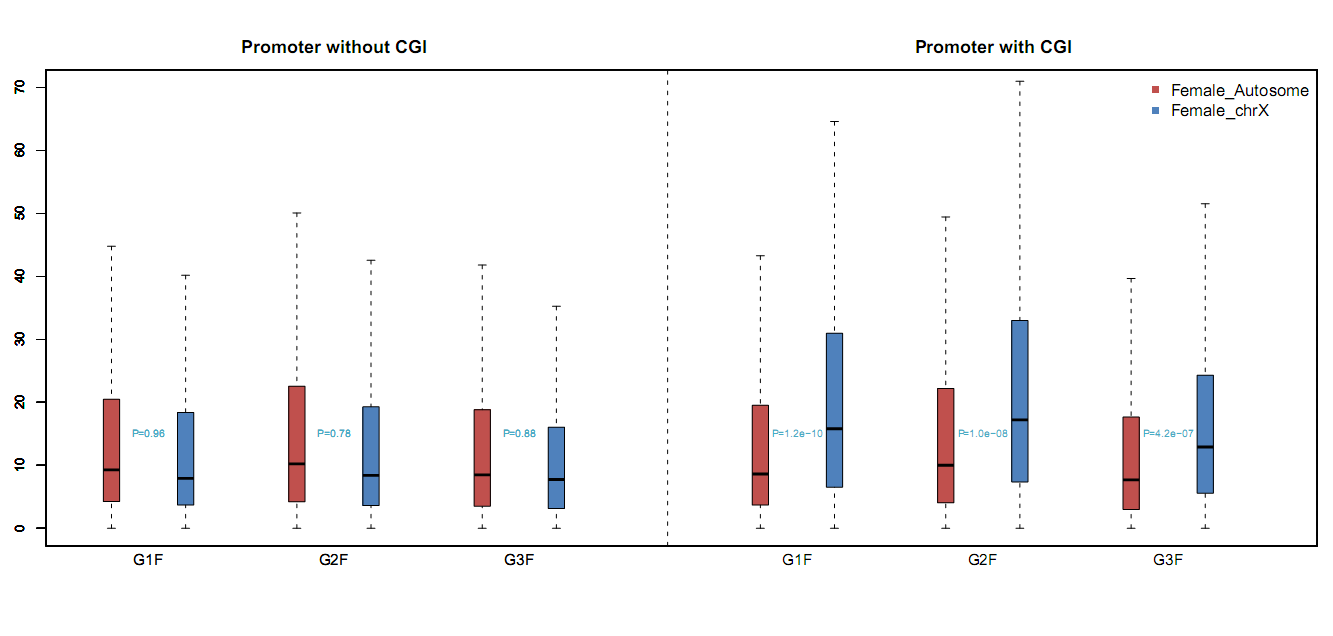


**Figure S2** Inactivation of gene promoters on the X chromosomes in females’ blood samples.


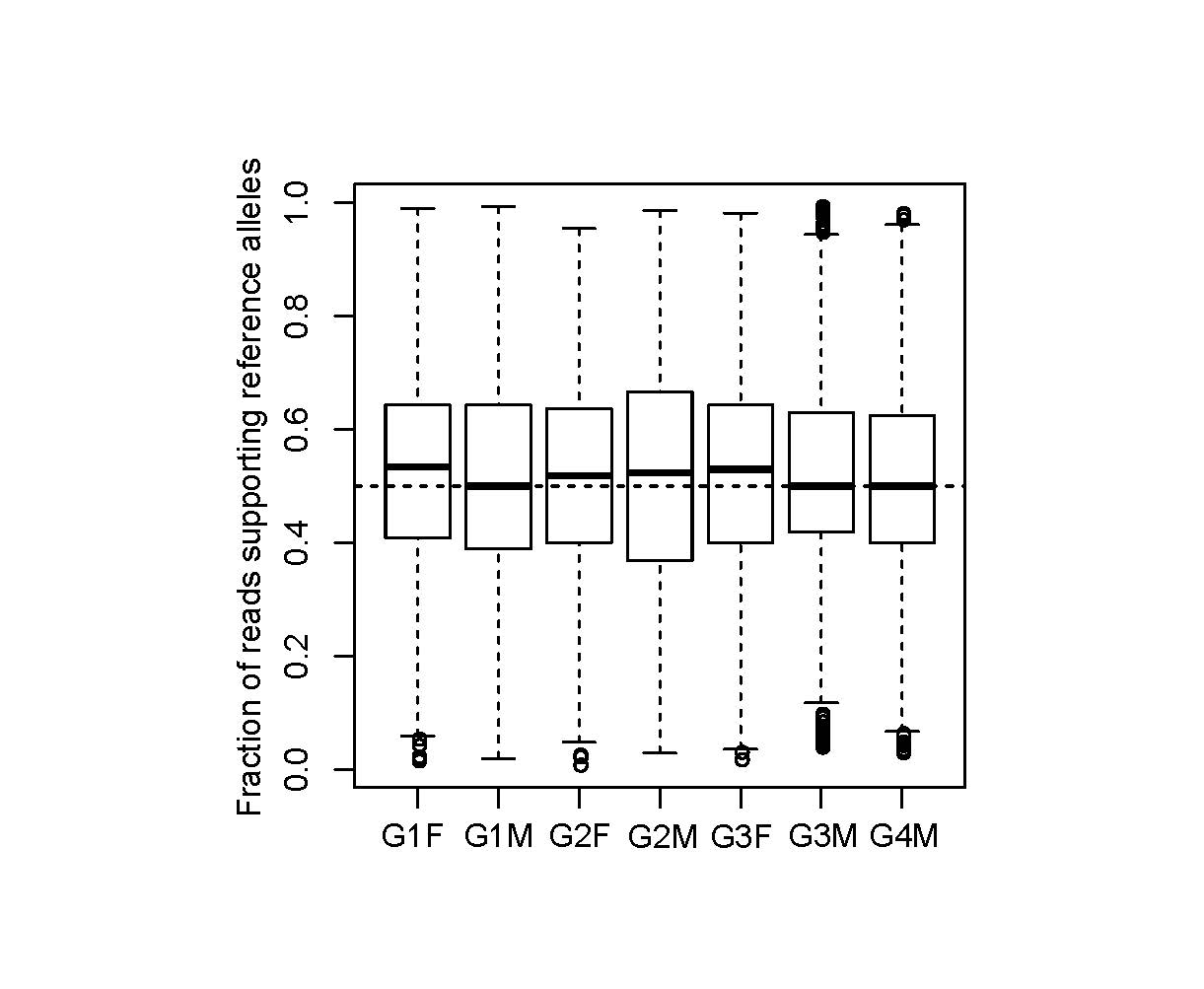


**Figure S3** Fractions of reads supporting reference alleles for the heterozygous non-CpG associated SNPs.


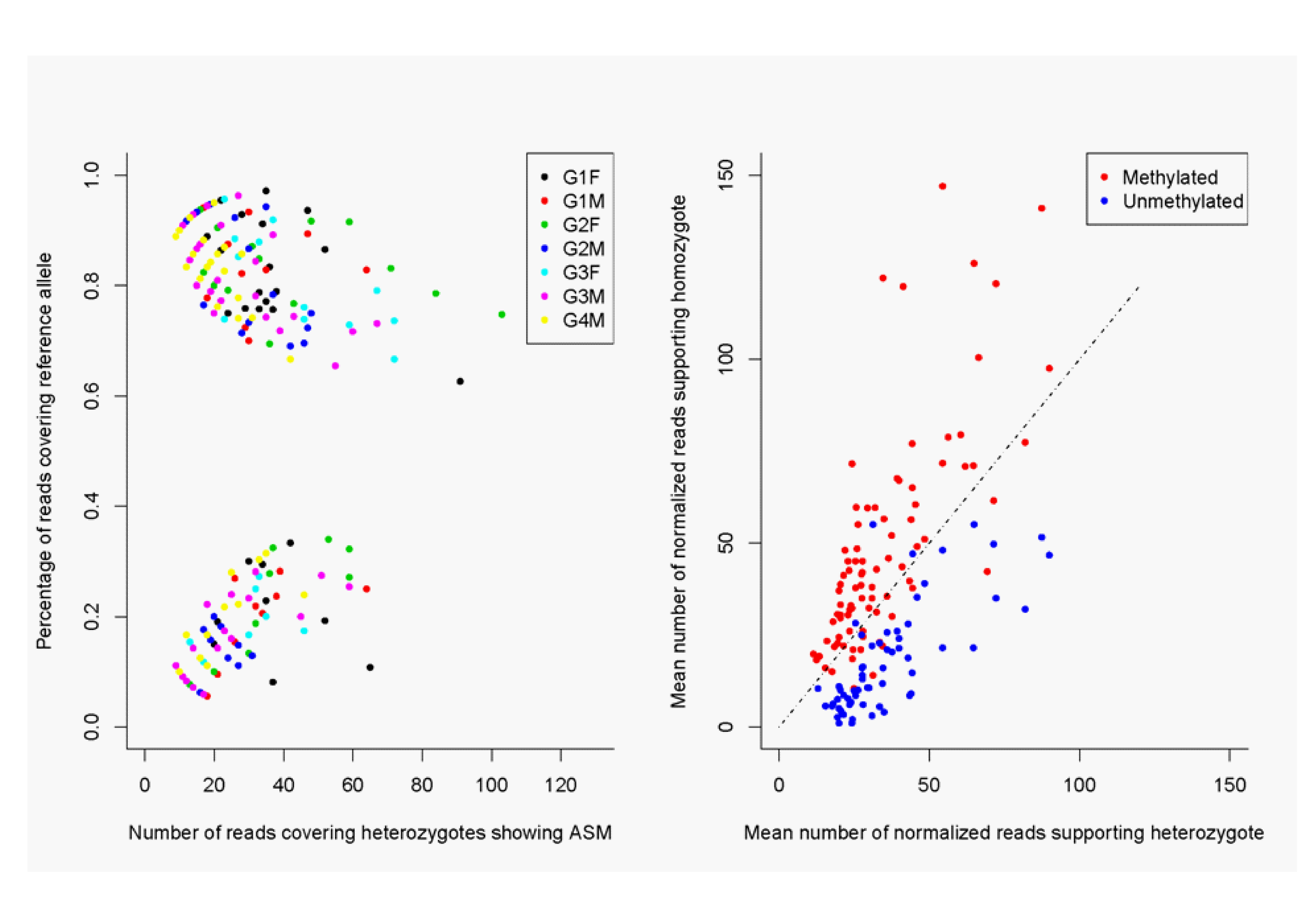


**Figure S4** The distribution pattern of the ASM associated tag SNPs. **(A)** Fraction of reads supporting the reference alleles of the ASM tag SNPs. **(B)** The mean numbers of reads supporting the different genotypes of the ASM tag SNPs.

**
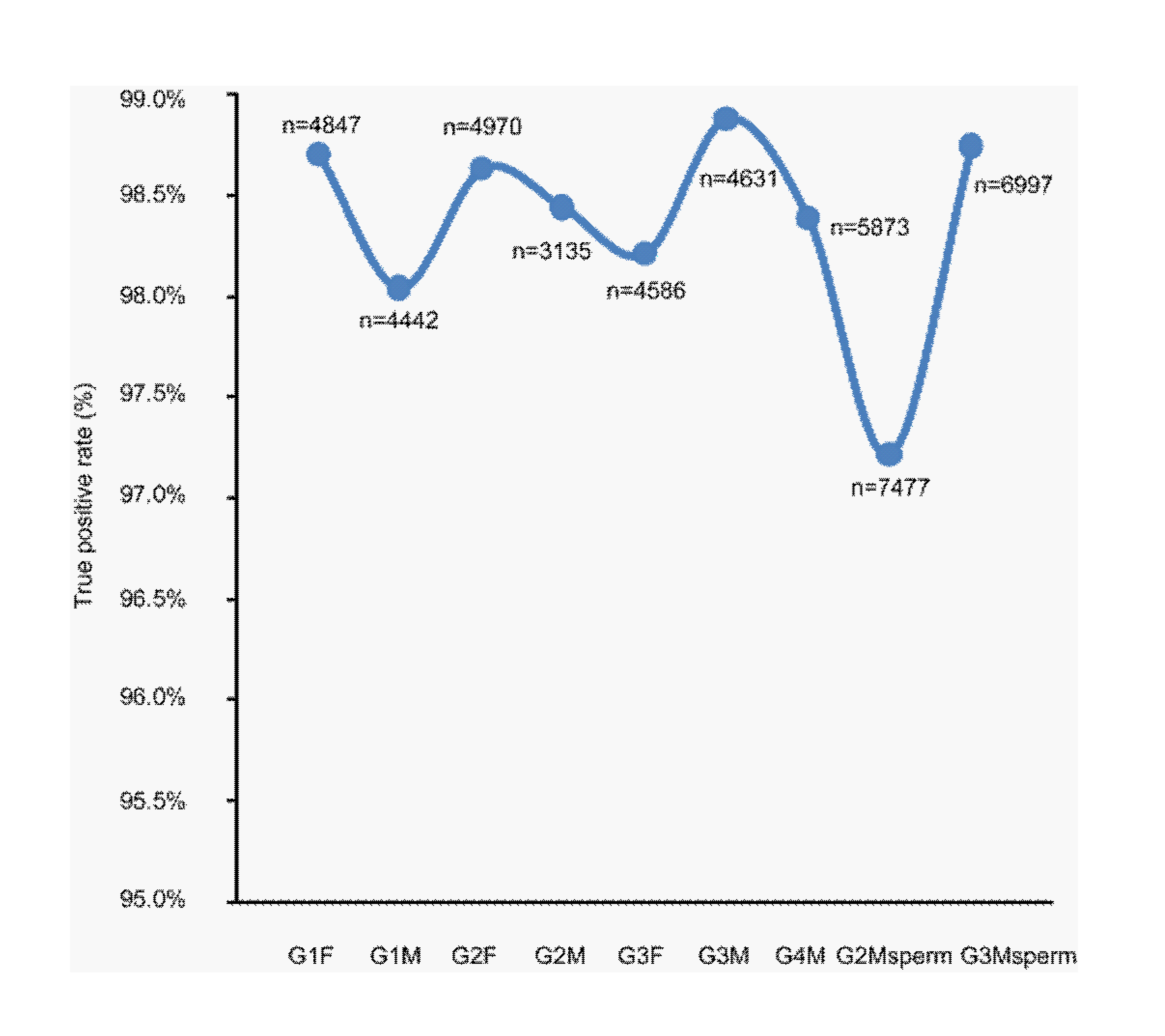
**

**Figure S5** Concordance rates between the genotypes called by MeDIP-seq and those generated by the Illumina SNP array.





**Figure S6** ASM events validated by Sanger sequencing of the cloned bisulfite PCR products. The sequencing reads are grouped according to the alleles at the tag SNP positions. The small squares represent the CpG sites on individual sequencing reads. The methylated CpGs are marked in dark red color, the unmethylated CpGs are marked in light blue color and CpGs affected by SNPs are marked in white color. The positions of the tag SNPs are also indicated. The distributions of the methylation rates of individual sequencing reads are shown in the box plots.


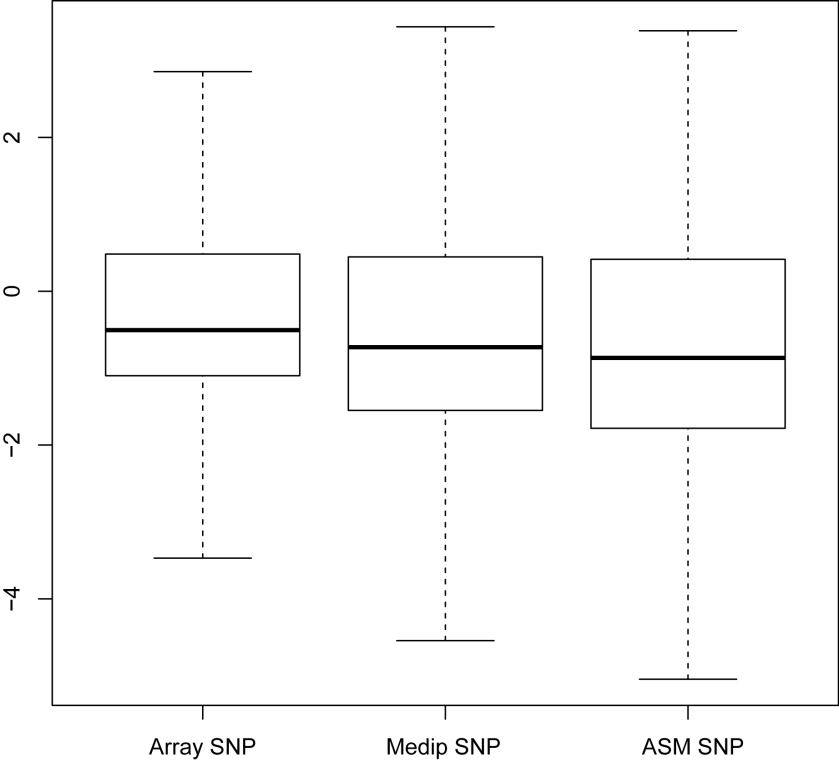


**Figure S7** The phyloP conservation scores for the ASM tag SNPs are significantly lower than other SNPs showing no allelic preference for DNA methylation. The midline represents the median conservation score and the box represents the 25% and 75% quartiles.





**Figure S8** Fraction of reads supporting the reference alleles of the ASM tag SNPs in the sperm samples.

**Table S1** The amount of sequence data generated for each sample in this study.

| **Sample ID** | **# of reads generated** | **# of bases generated** | **# of aligned reads** | **% of aligned reads** | **# of bases in aligned reads** | **# of effectively aligned reads** | **% of effectively aligned reads** | **# of bases in effectively aligned reads** |
| --- | --- | --- | --- | --- | --- | --- | --- | --- |
| **G1M** | 74,017,311 | 3,106,002,585 | 65,333,820 | 88.27% | 2,733,723,123 | 43,428,100 | 66.47% | 1,825,567,214 |
| **G2M** | 71,871,787 | 3,021,939,449 | 63,923,234 | 88.94% | 2,680,378,159 | 46,317,209 | 72.46% | 1,951,262,761 |
| **G3M** | 73,815,068 | 3,095,418,310 | 61,486,504 | 83.30% | 2,563,208,741 | 38,459,352 | 62.55% | 1,610,828,754 |
| **G4M** | 76,619,036 | 3,233,710,744 | 67,132,102 | 87.62% | 2,824,287,035 | 45,222,192 | 67.36% | 1,910,696,013 |
| **G2MSperm** | 74,766,955 | 3,143,708,429 | 69,324,214 | 92.72% | 2,909,135,102 | 51,641,006 | 74.49% | 2,176,305,520 |
| **G3MSperm** | 67,102,461 | 2,796,518,457 | 63,229,680 | 94.23% | 2,630,519,775 | 48,230,030 | 76.28% | 2,017,180,204 |
| **G1F** | 89,657,908 | 3,944,947,952 | 78,460,757 | 87.51% | 3,452,273,308 | 59,216,712 | 75.47% | 2,605,535,328 |
| **G2F** | 87,256,272 | 3,839,275,968 | 77,021,449 | 88.27% | 3,388,943,756 | 58,457,428 | 75.90% | 2,572,126,832 |
| **G3F** | 82,110,612 | 3,612,866,928 | 71,127,283 | 86.62% | 3,129,600,452 | 53,947,286 | 75.85% | 2,373,680,584 |

Reads were aligned to the UCSC reference human genome (hg18).

Table S2. Number of reads supporting the ASM events tagged by array SNPs in the blood samples

| Chr | Pos | G1F | G1M | G2F | G2M | G3F | G3M | G4M |
| --- | --- | --- | --- | --- | --- | --- | --- | --- |
| chr1 | 1488240 | 45,0 | 32,7 | 18,3 | 25,1 | 15,2 | 28,8 | 0,13 |
| chr1 | 8988719 | 44,0 | 41,0 | 25,11 | 17,0 | 65,1 | 28,1 | 47,0 |
| chr1 | 10402265 | 34,1 | 77,0 | 0,1 | 12,1 | 66,0 | 0,1 | 21,4 |
| chr1 | 91456236 | 16,2 | 14,2 | 14,3 | 12,1 | 31,0 | 30,0 | 9,0 |
| chr1 | 94369419 | 15,2 | 14,1 | 0,4 | 0,10 | 15,0 | 0,3 | 17,4 |
| chr1 | 204737450 | 44,3 | 21,1 | 0,9 | 23,0 | 37,3 | 22,3 | 0,2 |
| chr1 | 224884090 | 5,0 | 4,0 | 2,18 | 7,0 | 2,16 | 1,0 | 4,20 |
| chr1 | 226748372 | 1,39 | 0,64 | 77,0 | 0,46 | 37,13 | 61,32 | 46,0 |
| chr1 | 236156880 | 0,34 | 25,0 | 0,37 | 5,25 | 24,0 | 10,32 | 28,0 |
| chr10 | 13792387 | 9,21 | 12,41 | 20,0 | 2,23 | 16,0 | 28,0 | 20,0 |
| chr10 | 72494926 | 36,0 | 35,0 | 13,3 | 18,0 | 42,0 | 20,5 | 17,0 |
| chr10 | 72732054 | 3,34 | 27,0 | 10,0 | 5,38 | 9,0 | 23,0 | 38,0 |
| chr10 | 77343635 | 10,0 | 10,0 | 1,11 | 8,0 | 5,0 | 1,13 | 19,0 |
| chr10 | 118876328 | 30,6 | 61,0 | 48,0 | 28,11 | 58,0 | 62,0 | 31,0 |
| chr11 | 2500006 | 11,0 | 17,4 | 0,2 | 21,0 | 14,0 | 25,3 | 28,0 |
| chr11 | 8296639 | 0,19 | 22,0 | 0,33 | 4,23 | 22,0 | 6,23 | 5,0 |
| chr11 | 10742718 | 0,6 | 18,2 | 15,2 | 17,2 | 0,4 | 22,1 | 17,4 |
| chr11 | 44743582 | 3,0 | 14,0 | 1,12 | 1,0 | 5,0 | 1,22 | 8,0 |
| chr11 | 74519845 | 42,0 | 28,2 | 54,5 | 54,0 | 57,0 | 28,6 | 21,0 |
| chr11 | 78625273 | 27,0 | 45,0 | 18,1 | 14,0 | 4,0 | 17,1 | 19,0 |
| chr11 | 107107564 | 4,22 | 12,0 | 10,0 | 2,15 | 8,0 | 5,0 | 8,0 |
| chr11 | 116888904 | 13,0 | 18,0 | 27,0 | 11,0 | 11,1 | 22,0 | 12,1 |
| chr11 | 128317781 | 0,18 | 0,4 | 0,23 | 0,1 | 18,6 | 0,3 | 25,1 |
| chr12 | 129783263 | 23,0 | 55,0 | 17,0 | 8,0 | 13,2 | 40,0 | 17,4 |
| chr12 | 130844483 | 42,0 | 0,14 | 20,2 | 20,1 | 25,3 | 45,8 | 13,2 |
| chr12 | 131542948 | 0,16 | 30,12 | 0,18 | 18,4 | 0,21 | 47,18 | 0,20 |
| chr13 | 50261304 | 22,0 | 5,31 | 0,122 | 2,0 | 24,0 | 6,32 | 6,24 |
| chr13 | 84513855 | 22,0 | 22,0 | 24,0 | 14,0 | 11,1 | 17,0 | 10,1 |
| chr14 | 102668489 | 30,8 | 0,10 | 0,15 | 0,4 | 45,0 | 0,5 | 12,1 |
| chr14 | 102669064 | 14,28 | 0,34 | 0,43 | 0,15 | 47,0 | 0,59 | 15,32 |
| chr14 | 104266366 | 81,0 | 101,0 | 97,0 | 37,0 | 47,17 | 81,0 | 38,19 |
| chr14 | 105138663 | 3,0 | 30,4 | 4,0 | 14,2 | 2,0 | 28,0 | 15,0 |
| chr15 | 84099856 | 54,0 | 22,68 | 1,141 | 34,0 | 65,0 | 23,62 | 53,0 |
| chr16 | 1893096 | 3,0 | 10,0 | 1,11 | 4,0 | 2,13 | 3,17 | 2,13 |
| chr16 | 79730694 | 22,0 | 71,1 | 35,0 | 46,0 | 18,4 | 66,0 | 20,2 |
| chr16 | 83419235 | 14,3 | 0,11 | 40,0 | 12,1 | 26,4 | 10,0 | 23,0 |
| chr16 | 86246069 | 0,4 | 0,8 | 14,3 | 0,11 | 16,3 | 25,8 | 45,0 |
| chr16 | 87332684 | 0,1 | 0,8 | 15,1 | 0,1 | 16,0 | 22,5 | 28,0 |
| chr16 | 88190573 | 66,0 | 41,8 | 35,0 | 37,5 | 81,0 | 90,0 | 30,0 |
| chr17 | 787555 | 27,0 | 21,5 | 35,0 | 14,1 | 37,0 | 37,0 | 16,0 |
| chr17 | 21825882 | 33,0 | 20,5 | 0,22 | 24,0 | 47,0 | 34,3 | 36,0 |
| chr17 | 42150374 | 0,10 | 73,0 | 46,0 | 34,2 | 0,13 | 20,3 | 0,9 |
| chr17 | 64213939 | 26,2 | 0,10 | 56,0 | 12,1 | 45,0 | 15,0 | 39,0 |
| chr17 | 68246282 | 18,6 | 0,2 | 16,5 | 0,7 | 33,0 | 0,11 | 21,6 |
| chr18 | 466477 | 13,1 | 35,0 | 18,0 | 12,1 | 19,0 | 20,0 | 4,0 |
| chr18 | 41516357 | 22,7 | 53,0 | 37,0 | 21,4 | 25,3 | 40,0 | 24,0 |
| chr18 | 70793812 | 13,0 | 21,0 | 12,25 | 7,0 | 4,0 | 3,20 | 8,0 |
| chr19 | 2919157 | 25,8 | 20,5 | 19,5 | 24,0 | 0,6 | 25,0 | 25,5 |
| chr19 | 3689649 | 0,96 | 0,75 | 19,40 | 0,44 | 31,0 | 11,39 | 12,0 |
| chr19 | 48316888 | 91,0 | 156,0 | 44,4 | 126,0 | 106,0 | 44,1 | 27,4 |
| chr19 | 53812246 | 46,0 | 0,25 | 28,5 | 18,4 | 56,0 | 37,0 | 27,0 |
| chr2 | 1648126 | 0,51 | 0,63 | 18,35 | 0,43 | 39,0 | 0,47 | 13,31 |
| chr2 | 18429916 | 48,0 | 22,4 | 39,0 | 15,2 | 45,0 | 49,0 | 25,0 |
| chr2 | 25404634 | 16,0 | 35,0 | 19,2 | 12,0 | 21,0 | 27,8 | 16,2 |
| chr2 | 46262970 | 55,0 | 24,0 | 56,0 | 1,0 | 25,4 | 20,0 | 27,9 |
| chr2 | 69262726 | 4,17 | 17,0 | 0,55 | 4,0 | 7,31 | 3,17 | 9,0 |
| chr2 | 100376514 | 8,27 | 0,51 | 4,26 | 0,30 | 6,21 | 1,27 | 0,16 |
| chr2 | 108695786 | 77,0 | 30,11 | 83,0 | 18,5 | 80,0 | 34,0 | 24,0 |
| chr2 | 130234072 | 0,51 | 0,51 | 16,43 | 0,31 | 8,42 | 25,74 | 0,36 |
| chr2 | 219816544 | 6,0 | 58,0 | 33,0 | 51,0 | 32,4 | 81,0 | 32,5 |
| chr2 | 227701712 | 57,34 | 84,0 | 128,0 | 31,11 | 165,0 | 83,0 | 42,0 |
| chr2 | 236461287 | 21,1 | 45,1 | 27,4 | 44,0 | 18,4 | 90,0 | 24,4 |
| chr2 | 237922092 | 10,24 | 20,0 | 35,0 | 2,15 | 34,0 | 20,0 | 32,0 |
| chr2 | 238113746 | 0,27 | 0,24 | 4,0 | 0,17 | 11,0 | 1,17 | 2,19 |
| chr20 | 874585 | 0,49 | 37,0 | 34,0 | 5,33 | 36,0 | 15,39 | 34,0 |
| chr20 | 23430134 | 29,0 | 10,35 | 33,0 | 1,21 | 32,0 | 5,0 | 15,0 |
| chr20 | 37122642 | 1,54 | 15,40 | 24,0 | 4,20 | 0,104 | 11,30 | 0,43 |
| chr21 | 44240341 | 152,0 | 60,7 | 59,12 | 89,0 | 58,21 | 54,18 | 0,35 |
| chr21 | 44331247 | 10,0 | 12,34 | 0,55 | 7,0 | 0,40 | 6,35 | 0,24 |
| chr22 | 18061349 | 14,0 | 0,47 | 16,5 | 41,11 | 16,5 | 0,91 | 0,27 |
| chr22 | 25351425 | 4,0 | 1,24 | 2,41 | 2,0 | 0,35 | 5,30 | 9,24 |
| chr22 | 43186137 | 23,8 | 0,5 | 22,0 | 0,12 | 0,18 | 32,6 | 0,12 |
| chr22 | 47214755 | 43,0 | 48,17 | 69,0 | 41,18 | 54,0 | 113,0 | 75,0 |
| chr22 | 47215089 | 0,65 | 10,27 | 0,61 | 1,14 | 0,54 | 0,34 | 0,28 |
| chr3 | 50620417 | 28,9 | 75,15 | 71,0 | 0,20 | 58,15 | 44,15 | 0,23 |
| chr3 | 123486459 | 1,47 | 14,37 | 10,26 | 4,34 | 9,26 | 0,83 | 15,47 |
| chr3 | 128148697 | 0,43 | 147,0 | 33,10 | 48,18 | 0,47 | 0,57 | 0,45 |
| chr4 | 5729590 | 4,18 | 0,28 | 6,26 | 0,17 | 5,27 | 0,18 | 2,21 |
| chr4 | 147363022 | 10,42 | 53,0 | 25,0 | 4,30 | 12,0 | 27,0 | 23,0 |
| chr4 | 184516607 | 7,58 | 2,27 | 25,0 | 0,30 | 15,0 | 1,18 | 21,0 |
| chr5 | 11139565 | 19,3 | 0,1 | 0,1 | 0,0 | 37,0 | 0,0 | 16,2 |
| chr5 | 35962098 | 26,7 | 32,0 | 67,0 | 17,1 | 52,0 | 13,0 | 25,0 |
| chr5 | 179200389 | 37,0 | 30,12 | 27,0 | 46,20 | 0,9 | 105,0 | 20,4 |
| chr6 | 166746294 | 0,19 | 4,21 | 0,24 | 5,23 | 16,0 | 0,62 | 6,24 |
| chr6 | 167036150 | 26,0 | 0,7 | 0,3 | 17,4 | 0,6 | 23,3 | 0,8 |
| chr7 | 188894 | 0,55 | 101,0 | 77,26 | 51,17 | 151,0 | 42,11 | 28,8 |
| chr7 | 48420703 | 0,60 | 10,38 | 2,102 | 12,33 | 0,99 | 15,61 | 0,54 |
| chr7 | 49854295 | 0,2 | 0,2 | 0,7 | 0,1 | 18,4 | 0,1 | 16,1 |
| chr7 | 55189149 | 0,36 | 0,74 | 126,0 | 0,30 | 53,26 | 73,28 | 69,0 |
| chr7 | 100140370 | 15,0 | 17,0 | 9,1 | 17,0 | 14,0 | 18,3 | 17,0 |
| chr7 | 154305587 | 31,3 | 22,1 | 68,0 | 0,4 | 45,0 | 56,6 | 17,4 |
| chr7 | 155067461 | 19,4 | 25,1 | 51,0 | 15,1 | 37,0 | 30,3 | 10,0 |
| chr8 | 1118309 | 27,8 | 40,2 | 57,0 | 0,0 | 7,26 | 78,0 | 31,10 |
| chr8 | 1137989 | 76,0 | 0,32 | 66,18 | 47,2 | 102,1 | 83,30 | 54,0 |
| chr8 | 2193996 | 26,0 | 38,30 | 21,0 | 11,1 | 24,1 | 44,0 | 27,9 |
| chr8 | 140283197 | 45,7 | 0,22 | 77,0 | 0,5 | 38,12 | 25,6 | 0,17 |
| chr8 | 142272066 | 0,28 | 0,37 | 0,23 | 0,34 | 8,26 | 0,37 | 1,12 |
| chr9 | 130438457 | 3,17 | 0,64 | 0,33 | 0,21 | 2,12 | 0,52 | 8,28 |
| chr9 | 135656304 | 21,8 | 27,0 | 27,0 | 15,2 | 29,0 | 35,0 | 34,0 |
| chr9 | 136893330 | 25,0 | 40,0 | 16,4 | 44,0 | 40,0 | 20,1 | 17,0 |

For each array SNP, the numbers before and after the comma (,) show the number of reads supporting the reference and variant allele, respectively. Individuals who were genotyped as heterozygotes by the SNP array at the ASM loci were highlighted by yellow color.
